# Influence of Race on Microsatellite Instability and CD8^+^ T Cell Infiltration in Colon Cancer

**DOI:** 10.1371/journal.pone.0100461

**Published:** 2014-06-23

**Authors:** John M. Carethers, Bhavya Murali, Bing Yang, Ryan T. Doctolero, Akihiro Tajima, Ranor Basa, E. Julieta Smith, Monte Lee, Ryan Janke, Tina Ngo, Ruth Tejada, Ming Ji, Matthew Kinseth, Betty L. Cabrera, Katsumi Miyai, Temitope O. Keku, Christopher F. Martin, Joseph A. Galanko, Robert S. Sandler, Kathleen L. McGuire

**Affiliations:** 1 Division of Gastroenterology, Department of Internal Medicine, University of Michigan, Ann Arbor, Michigan, United States of America; 2 Department of Biology, Molecular Biology Institute, San Diego State University, San Diego, California, United States of America; 3 Graduate School Public Health, San Diego State University, San Diego, California, United States of America; 4 Department of Medicine, University of California San Diego, San Diego, California, United States of America; 5 Department of Pathology, University of California San Diego, San Diego, California, United States of America; 6 Moores Cancer Center, University of California San Diego, San Diego, California, United States of America; 7 Department of Medicine, University of North Carolina, Chapel Hill, North Carolina, United States of America; 8 Department of Epidemiology, University of North Carolina, Chapel Hill, North Carolina, United States of America; Howard University, United States of America

## Abstract

African American patients with colorectal cancer show higher mortality than their Caucasian counterparts. Biology might play a partial role, and prior studies suggest a higher prevalence for microsatellite instability (MSI) among cancers from African Americans, albeit patients with MSI cancers have improved survival over patients with non-MSI cancers, counter to the outcome observed for African American patients. CD8^+^ T cell infiltration of colon cancer is postively correlated with MSI tumors, and is also related to improved outcome. Here, we utilized a 503-person, population-based colon cancer cohort comprising 45% African Americans to determine, under blinded conditions from all epidemiological data, the prevalence of MSI and associated CD8^+^ T cell infiltration within the cancers. Among Caucasian cancers, 14% were MSI, whereas African American cancers demonstrated 7% MSI (*P* = 0.009). Clinically, MSI cancers between races were similar; among microsatellite stable cancers, African American patients were younger, female, and with proximal cancers. CD8^+^ T cells were higher in MSI cancers (88.0 vs 30.4/hpf, *P*<0.0001), but was not different between races. Utilizing this population-based cohort, African American cancers show half the MSI prevalence of Caucasians without change in CD8^+^ T cell infiltration which may contribute towards their higher mortality from colon cancer.

## Introduction

Mortality from colon cancer is higher in African Americans than Caucasians at every stage of disease [1], [2]. While the mortality difference could be in part related to clinical care or comorbidity, it could also have contributions from differences from within the cancer [3]. Prior studies have had insufficient numbers of African Americans to adequately examine racial differences from tumor characteristics that could influence mortality [4], [5].

Microsatellite instability (MSI) is a genetic biomarker of hypermutable colon cancers, driven by inactivation of the DNA mismatch repair (MMR) system [6], [7]. Its presence depicts a better outcome for patients when compared to same-staged colon cancer patients without MSI [3]. This may be due in part to neoantigens generated by mutational frameshifts within coding regions of specific genes as a consequence of inactivation of DNA MMR in the colon cancer epithelial cell, attracting specific immune cells that can help contain the tumor and limit metastasis [7], [8]. In particular, MSI colon cancers are associated with increased intraepithelial CD8^+^ T cells when compared to microsatellite stable (MSS) cancers [8]–[10], and many MSI cancers demonstrate a “Crohn's-like” lymphoid “granuloma” reaction histologically within the epithelial component of the adenocarcinoma [7]–[9]. MSI cancers comprise 15–20% of all colon cancers, have diploid cells and are inversely related to allelic loss. MSI cancers are more likely to have mucinous features and poor differentiation at histology, and ∼70% are located proximal to the splenic flexure within the colon. MSI cancers may progress more rapidly than MSS cancers [7], [11], and may drive sessile serrated adenomas into cancers [12], [13]. Additionally, patients with stage II/III MSI cancers do not improve their survival with 5-fluorouracil-based chemotherapy [14], [15].

Colon cancers from African Americans share some clinical and epidemiological features with MSI cancers, but not others. For instance, African Americans have more propensity for proximal colon cancers and polyps, and tumor progression may be more rapid based on the younger age for cancer diagnosis [1], [2], [16]. Some reports suggest more mucinous features anectdotally in African American colon cancers [1]. These features would suggest the prevalence of MSI would be relatively higher in African American colon cancers. Conversely, African Americans tend to present with advanced stage of disease, and possess poor 5-year survival in contrast to that of patients with MSI cancers [1]. These features would suggest MSI would be relatively lower in African American colon cancers. Evaluation of African American colon cancers for MSI has been sparse with only two groups having published data, one showing an MSI frequency of 23% (7 of 31 samples) and the other with 48% (10 of 21 samples) among African American cancers [4], [5], suggesting a higher-than-average frequency. Both groups utilized stored samples with linked racial data; neither are studies from within a population. In the present study, we utilized a population-based colon cancer cohort to more accurately determine the frequency of MSI in African American colon cancers. Our data indicate that there is half the frequency of MSI among African American colon cancers compared to Caucasian colon cancers, suggesting that once an African American is diagnosed with colon cancer, the benefit from improved survival associated with MSI cancers is more limited in this population.

## Materials and Methods

### Patients and Specimens

Data were collected as part of the North Carolina Colon Cancer Study (NCCCS), a population-based, case–control study conducted in 33 contiguous counties of central and eastern North Carolina, including both rural and urban areas [17]. The counties were selected to provide adequate representation by African American and rural residents, and to avoid referral or selection patterns that would impede case ascertainment and/or compliance. The Institutional Review Board of the University of North Carolina School of Medicine approved the original study protocol.

Cases were identified between October 1, 1996 and September 1, 2000 using a rapid ascertainment system implemented in cooperation with the North Carolina Central Cancer Registry. Eligible cases were between the ages of 40 and 80 yr, had pathologically confirmed invasive adenocarcinoma of the colon, and resided in the 33-county area. All eligible African American patients were invited to participate. A random sample of Caucasian patients of similar age (±5 yr) and sex was drawn to provide an equal number of African American and Caucasian cases. Controls younger than 65 years from the same 33-county area were selected from the Department of Motor Vehicles Registry database, and controls 65 years and older were selected from Health Care Financing Administration database.

Of 935 potential colon cancer cases identified (464 African American, 471 Caucasian), physicians refused access for 31 African Americans and 35 Caucuasians, contact was incomplete for 29 African Americans and 29 Caucasians, and 81 African Americans and 45 Caucasians refused to participate. The overall cooperation rate (interviewed/interviewed + refused) was 84%: 79% for African Americans, 89% for Caucasians. A further 33 patients were excluded because the quality of the interview was judged inadequate. Consequently, there were 643 cases: 294 African Americans and 349 Caucasians. The overall cooperation rate for controls was 62%: 59% for African Americans and 65% for Caucasians; 1048 controls, 437 African American and 611 Caucasian participants.

Cases and controls were selected using randomized recruitment to achieve an age, race, and sex ratio optimized for statistical efficiency. African American cases were oversampled to yield a 1∶1 Caucasian:African American ratio, and controls were sampled to match the distribution by race, age, and gender of the cases.

Tissue samples were collected at the time of surgery and fixed in formalin. Archived formalin-fixed, paraffin-embedded (FFPE) tissue was provided for DNA isolation and immunohistochemistry (IHC) in the studies described here. There were 503 total cases with available tissue for analysis for this study. The University of California San Diego, San Diego State University, and the University of Michigan Human Subjects Committees approved the research utilizing these existing pathological specimens.

### DNA Extraction and Microsatellite Analysis

DNA was extracted from formalin-fixed, paraffin-embedded tissues of each patient's colorectal tumor and surrounding normal tissue [14]. Each area microdissected was identified by the pathologist on a reference hematoxylin and eosin stained slide. Unstained tissue slices adjacent to the reference slide were microdissected according to areas identified on the reference slide using a surgical scalpel blade. The dissected specimen was deparaffinized in a microfuge tube with xylene, and the DNA was purified with ethanol and GeneReleaser (BioVentures, Murfeesboro, TN) according to the manufacturer's recommendations. Subsequently, the samples were treated with 200 µg/ml of proteinase K (Sigma, St. Louis, MO) and incubated at 55°C for 5 hours. The proteinase K was destroyed by heating the sample to 95°C for 15 minutes, and the samples were immediately iced and stored for polymerase chain reaction (PCR) analysis.

For determination of MSI, we used the NCI-recommended panel of 5 microsatellite markers (BAT25, BAT26, D5S346, D2S123, and D17S250) to classify the tumor as MSI-High (associated with inactivation of DNA MMR), and MSI-Low or microsatellite stable (MSS) which are not associated with DNA MMR inactivation. MSI-High was defined as 2 or more markers demonstrating novel alleles compared to non-tumor tissue, MSI-L as 1 marker with a novel allele, and MSS as no marker with novel alleles. Because of the similar features of MSI-Low and MSS tumors [7], these two groups were included together in our cohort. One primer from each microsatellite marker pair was radio-endlabeled with ^32^P or fluorescently labeled. PCR was carried out on the microdissected template DNA in a reaction containing 0.125 pmoles each of the endlabeled and “cold” primers, 0.25 U of Taq DNA polymerase, 40 µM of dNTP stock solution, and final concentration of 1.5 to 2.0 µM magnesium. PCR products were denatured in 95% formamide, and electrophoresed on a 6% polyacrylamide gel containing 7.5 M urea. The gels were then dried, and exposed by autoradiography to x-ray film or in a phosphorimager (Molecular Dynamics, Sunnyvale CA). Alternatively, PCR reactions utilizing fluorescently-labeled primers were sent directly for DNA sequencing.

Genetic analyses were blinded and carried out separately and independently from the epidemiological data collected in the study.

### Immunohistochemistry and CD8^+^ T Cell Counts

To perform immunohistochemistry for CD8^+^ T cells, formalin-fixed, paraffin-embedded samples were first deparaffinized in xylene and rehydrated. Endogenous peroxide activity was blocked with 3% hydrogen peroxide for 20 min. Antigen retrieval was performed in the microwave for 10 minutes in 10 mM Citrate (pH = 6.0) and 0.05% Tween 20. After cooling, non-specific protein binding was blocked with 1% BSA in PBS, and monoclonal mouse anti-human CD8 antibody (DakoCytomation, Clone C8/144B, Carpinteria, CA) was added overnight at 4°C. Cells were visualized using the DakoCytomation LSAB2 System-HRP kit, following the manufacturer's directions. The slides were counterstained with Mayer's hematoxylin, rinsed with ammonium hydroxide, dried and coverslipped. The samples were then analyzed by light microscopy.

For cell counts, each slide was evaluated at low power (50X) for the areas that had the highest concentration of stained cells. Three high-powered fields (hpf = 100X) were photographed and intraepithelial CD8^+^ T cells were counted independently in each hpf by two observers. If counts showed discrepancies >10%, the two observers evaluated the photograph together and then re-counted independently. The results from the two observers were averaged.

### Statistical Analysis

Analyses were conducted using SAS (version 9.1; SAS Institute, Inc., Cary, NC) and based on 2-sided *P* values. Statistical analyses of descriptive values were as follows: for continuous variables, the T-test; for categorical and pathological values, Fisher's Exact Test. We calculated adjusted odds ratios (OR) and 95% confidence intervals (95% CI) using unconditional logistic regression models (using the Proc Logistic module of SAS). The following covariates were considered for inclusion in the multivariate models for association with MSI cancers: age (continuous), gender (male, female), cancer stage (classified at time of surgery), race (African American, Caucasian), mucinous tumor (yes, no), tumor differentiation, inflammatory infiltrate, and cancer location in the colon (right-sided defined as proximal to the splenic flexure). Significant interaction was seen between race and tumor site, and there was weak association interaction between MSI status and age. Models constructed show both of those interactions.

Data obtained on CD8^+^ T cell counts/hpf in the total samples analyzed did not follow a normal, Gaussian distribution; therefore, non-parametric statistical tests were required to evaluate the significance of any difference between two groups in each of the analyses. The Mann-Whitney *U* test was performed to determine whether any two independent sample sets had data distributions that differed significantly. Statistical analyses for CD8^+^ T cell counts were performed using *IBM SPSS Statistics*, version 21.0.0.0 (IBM Corporation, 2012).

*P* values<0.05 were considered statistically significant.

## Results

We utilized a 503-person, true population-based colon cancer cohort with prospective collection of data from 33 counties in North Carolina that was designed to oversample African Americans in the cohort (the North Carolina Colon Cancer Study, NCCCS) [17]. It is important to note that the MSI analyses were blinded and carried out separately and independently from the epidemiological data collected in the study. Among the 503 colon cancers, 227 (45%) were from African Americans, and 276 (55%) were from Caucasians. Overall, 54 (11%) cancers demonstrated MSI. However, the prevalence of MSI in the cancers differed between African Americans and Caucasians. There were 39/276 (14%) Caucasians with MSI cancers, compared to 15/227 (7%) African Americans with MSI cancers (**Table 1** and **Figure S1**). Compared to MSS cancers, MSI cancers were associated with Caucasian patients (*P* = 0.009), a mucinous histology (*P* = 0.0004), poor differentiation (*P* = 0.03), a “Crohn's-like” lymphoid and intraepitheilial lymphocytic reaction (*P* = 0.0001), and location in the proximal colon (*P*<0.0001) (**Table 1**). There was no difference in age, gender, or cancer stage between patients in the MSI and MSS groups. Except for the racial difference, the findings associated with MSI tumors were previously known and serve to validate our data. We performed stepwise regression assessing all clinicopathological associations, and race, inflammatory infiltrate, and tumor site remained in the final model. There was significant interaction between race and tumor site. Patients with MSI cancers have an odds ratio (OR) of 3.21 for the presence of Crohn's like histology, and are less likely to be African Americans (with either proximal cancers, OR = 0.26, or distal cancers, OR = 0.16) or Caucasians with distal cancers (OR = 0.12) (**Table 2**). Adding age, which was weakly related to MSI but not race, did not significantly alter these odds ratios in the stepwise regression model.

**Table 1:** MSI vs. MSS clinicopathological associations between African Americans and Caucasians in the NCCCS.

	MSI	MSS	*P* value	CA MSI	AA MSI	*P* value	CA MSS	AA MSS	*P* value
	(*N = 54*)	(*N = 449*)		(*N = 39*)	(*N = 15*)		(*N = 237*)	(*N = 212*)	
Mean Age (S.E.), years	66.2 (1.4)	63.7 (0.5)	0.10	67.8 (1.6)	61.9 (3.0)	*0.07*	64.8 (0.6)	62.6 (0.7)	**0.02**
Sex, *N* (%): Male	22 (41)	229 (51)	0.19	19 (49)	3 (20)	*0.07*	136 (57)	93 (44)	**0.008**
Female	32 (59)	220 (49)		20 (51)	12 (80)		101 (43)	119 (56)	
Cancer Stage, *N* (%)			0.22			0.28			0.96
Local	21 (39)	166 (37)		18 (45)	3 (23)		86 (36)	80 (37)	
Regional	32 (59)	238 (53)		20 (52)	12 (77)		128 (54)	110 (52)	
Distant	1 (2)	45 (10)		1 (3)	0 (0)		23 (10)	22 (11)	
Race, *N* (%): Caucasian	39 (72)	237 (53)	**0.009**	**-**	**-**	**-**	**-**	**-**	**-**
African American	15 (28)	212 (47)							
Tumor, *N* (%): Non-Mucinous	37 (69)	400 (89)	**0.0004**	27 (70)	10 (67)	1	211 (89)	189 (89)	1
Mucinous	17 (31)	49 (11)		12 (30)	5 (33)		26 (11)	23 (11)	
Differentiation, *N* (%): Well	0 (0)	13 (3)	**0.03**	0 (0)	0 (0)	0.74	7 (3)	6 (3)	0.74
Moderate	36 (67)	355 (79)		27 (68)	9 (62)		191 (80)	164 (78)	
Poor	18 (33)	81 (18)		12 (32)	6 (38)		39 (17)	42 (20)	
Infiltrate, *N* (%): lymphocytic	25 (46)	332 (74)	**0.0001**	19 (47)	6 (42)	1	167 (71)	165 (79)	0.10
Crohn's-like	29 (54)	117 (26)		20 (53)	9 (58)		70 (29)	47 (21)	
Site, *N* (%): Left colo	10 (19)	229 (51)	**<0.0001**	5 (13)	5 (33)	0.12	132 (56)	97 (46)	**0.04**
Right colon	44 (81)	220 (49)		34 (87)	10 (67)		105 (44)	115 (54)	

CA = Caucasian American; AA = African American. Lymphocytic pattern refers to infiltrate at the interface of the normal bowel wall and tumor. Crohn's-like pattern refers to lymphoid and granuloma-like clusters in the tumor, and intraepithelial lymphocytes.

**Table 2:** Results of stepwise regression model for co-variables for MSI versus MSS cancers.

Infiltrate	Odds Ratio for MSI Cancer (without age in model)	Confidence Intervals	Odds Ratio for MSI Cancer (with age in model)	Confidence Intervals
Lymphocytic	1.0	referent	1.0	referent
Crohn's-like	3.21	1.68, 6.13	3.19	1.67, 6.09
Race-Site				
CA-Right	1.0	referent	1.0	referent
CA -Left	0.12	0.04, 0.33	0.12	0.05, 0.33
AA-Right	0.26	0.11, 0.60	0.26	0.11, 0.61
AA-Left	0.16	0.05, 0.48	0.17	0.06, 0.51

CA = Caucasian American; AA = African American; Right = colon proximal to and including the splenic flexure; Left = colon distal to the splenic flexure.

We assessed whether there were any differences between races within MSI or MSS cancer groups. We found no difference between African American and Caucasian patients with MSI cancers, although there was a trend for African American cancers to be from younger and female patients (**Table 1**). Among MSS cancers, African American patients were significantly younger (62.6 vs 64.8 years, *P* = 0.02), more likely female (56% vs. 43%, *P* = 0.008), and more likely to have a proximal colon tumor (54% vs. 44%, *P* = 0.04) (**Table 1**). Our data regarding African American MSS cancers is consistent with African American females possessing more advanced adenomatous polyps [18], and SEER and American Cancer Society data consistently showing earlier age and higher prevalence of proximal colon cancers among African American patients compared to Caucasian patients [1], [2].

**Figure 1:**
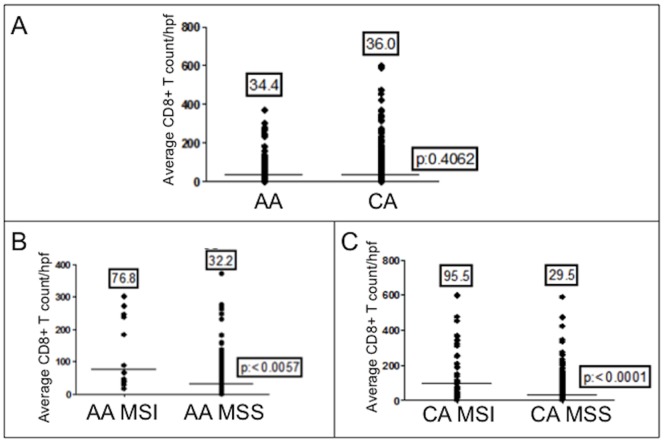
CD8^+^ T cell infiltration in colon cancers. (A) Immunohistochemistry of CD8^+^ T cells within malignant epitheilial glands of a colon cancer. Note the presence of intraepithelial CD8^+^ T cells (*inset*). (B) CD8^+^ T cell counts between MSI and MSS cancers. (C) CD8^+^ T cell counts of MSI cancers between races. (D) CD8^+^ T cell counts of MSS cancers between races. Note there is no difference between African Americans and Caucasians comparing MSI or MSS cancers. The number above each dot blot are means; the horizontal bar represents the mean number among the cancers.

**Figure 2:**
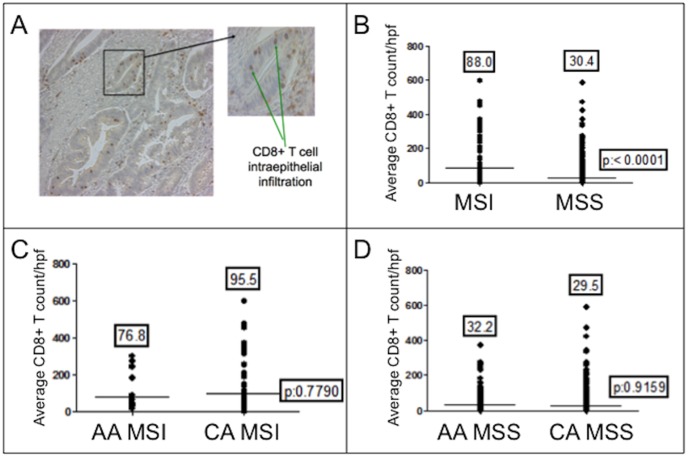
CD8^+^ T cell infiltration in colon cancers. (A) CD8^+^ T cell counts between races, regardless of microsatellite instability. (B) CD8^+^ T cell counts between MSI and MSS cancers from African Americans. (C) CD8^+^ T cell counts between MSI and MSS cancers from Caucasians. The number above each dot blot are means; the horizontal bar represents the mean number among the cancers.

We were able to determine the number of intraepithelial CD8^+^ T cells within 475 cancers to discern any difference between races, as higher numbers have been associated with MSI cancers [9] and improved survival [3], [19]. MSI cancers had higher mean CD8^+^ T cell counts when compared to MSS cancers (88.0 vs. 30.4/hpf, *P*<0.0001) (**Figure 1**), and this difference was present between MSI and MSS cancers from African American patients (76.8 vs 32.2/hpf, *P* = 0.0057) and from Caucasian patients (95.5 vs. 29.5/hpf, *P*<0.0001) (**Figure 2**). Among MSI cancers, there was no difference in mean CD8^+^ T intraepithelial cell infiltration between African Americans and Caucasians; likewise, there was no difference between races among MSS cancers (**Figure 1**). Our data is consistent with an association between CD8^+^ T cell intraepithelial infiltration and MSI cancers. While we observed no difference for overall mean CD8^+^ T cell counts between races (**Figure 2**), the mean CD8^+^ T cell count was consistently lower in African Americans compared to Caucasians (except for MSS cancers), with little or no representation in the upper quartile of CD8^+^ T cell counts (**Figure 2A** and **Figure 1BC**). Thus, cancers with very high infiltration of CD8^+^ T cells were more often and in some comparisons almost exclusively from Caucasian patients.

## Discussion

This population-based study demonstrates the novel finding that colon cancers from African American patients have half the frequency of MSI compared to Caucasians, without apparent change in the extent of intraepithelial CD8^+^ T cell infiltration. This finding is in contrast with two other studies [4], [5] that were not population-based and which utilized stored samples with linked racial data, and we believe that this study design difference is the reason for the contrasting results between studies. As MSI is a good prognostic marker when compared to same-staged patients with MSS tumors [3], the lesser frequency of MSI among African American colon cancers suggests that this may in part contribute towards the higher mortlity from colon cancer observed in this racial group [1], [2], [20].

The near absence of very high CD8^+^ T cell counts among African American cancers might suggest an alteration in immune function, and this will need to be explored further. However, we identified no differences when comparing the mean CD8^+^ T cell infiltration counts between races for MSI cancers or MSS cancers, suggesting that race *per se* is not a determinant of CD8^+^ T cell infiltration. Our data confirms the strong association between CD8^+^ T cell infiltration and the MSI status of a colon cancer, consistent with the notion that neoantigens driven by DNA MMR deficiency and subsequent frameshift mutation of target genes is operative [8], as well as CD8^+^ T cells are associated with improved survival from colon cancer [19]. The strong link between MSI status and CD8^+^ T cell infiltration might suggest that CD8+ T cells are the reason for the observed improved outcome for MSI tumors. For instance, patients with MSI colon cancers do not benefit from 5-FU chemotherapy in several studies, and 5-FU treatment might even decrease survival in these patients [14], [15]. It may be that adenocarcinoma cells with defective DNA MMR function are resistant to 5-FU, but that the CD8^+^ T cells are sensitive to 5-FU, with 5-FU treatment killing the beneficial effect of the CD8^+^ T cell infiltration [13]. The absence of very high CD8^+^ T cells among African American cancers might simulate a similar effect as theorized for 5-FU treatment of patients with MSI cancers.

Another biomarker of defective DNA MMR in sporadic colorectal cancers is elevated microsatellite alterations at selected tetranucelotide repeats, or EMAST, found in up to 60% of colorectal cancers and caused by somatic inactivation of hMSH3 [21], [22]. EMAST is associated with metastasis and poor prognosis [23], [24] and appears to be twice as common among rectal cancers from African Americans compared with Caucasians [24]. Thus, the contribution of EMAST, a poor prognosticator for patients with colon cancer, coupled with reduced prevalence of MSI, a good prognosticator, together make up two biological processes involved with inflammation and defects of DNA MMR that may worsen the outcome of African American patients with colon cancer.

Our data also demonstrate the predominant proximal nature of colon cancer in African Americans that is not due to MSI. Or data is consistent with prior SEER and American Cancer Society data [1], but highlight the right-sided nature of cancers in the absence of MSI tumors. Why there are more proximal colon cancers in African American patients is not known. However, this has implications for the approach to colon cancer screening in this population.

In conclusion, we find half the prevalence of MSI among African American patients with colon cancer as compared to Causcasian patients without change in CD8^+^ T cell infiltration. This finding may contribute towards African Americans' higher mortality from colon cancer.

## Supporting Information

Figure S1 Distribution of MSI and MSS tumors among African American and Caucasian patients in the NCCCS.
(TIFF)
