# Correction: Influence of Race on Microsatellite Instability and CD8^+^ T Cell Infiltration in Colon Cancer

**DOI:** 10.1371/journal.pone.0103699

**Published:** 2014-07-21

**Authors:** The PLOS ONE Staff

The images for Figure 1 and Figure 2 were inadvertently swapped. Please view the correct images and legends for Figure 1 and Figure 2.

**Figure 1:**
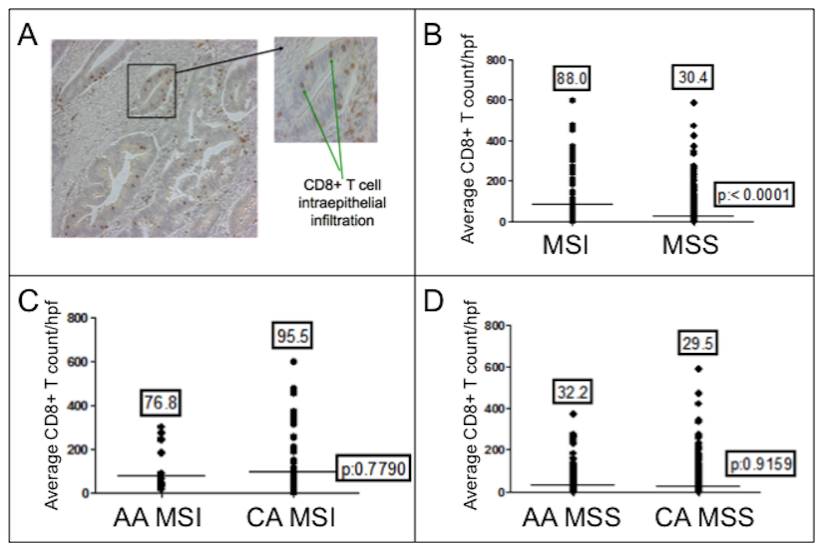
CD8^+^ T cell infiltration in colon cancers. (A) Immunohistochemistry of CD8^+^ T cells within malignant epitheilial glands of a colon cancer. Note the presence of intraepithelial CD8^+^ T cells (*inset*). (B) CD8^+^ T cell counts between MSI and MSS cancers. (C) CD8^+^ T cell counts of MSI cancers between races. (D) CD8^+^ T cell counts of MSS cancers between races. Note there is no difference between African Americans and Caucasians comparing MSI or MSS cancers. The number above each dot blot are means; the horizontal bar represents the mean number among the cancers.

**Figure 2:**
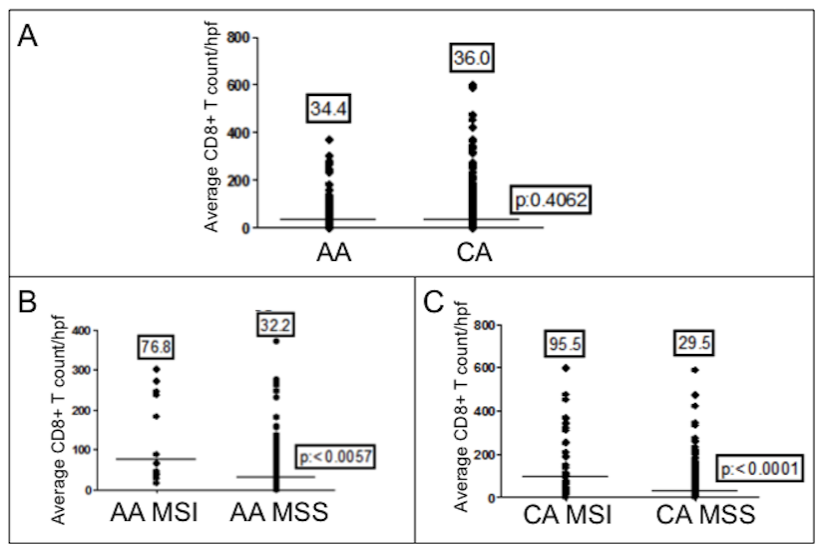
CD8^+^ T cell infiltration in colon cancers. (A) CD8^+^ T cell counts between races, regardless of microsatellite instability. (B) CD8^+^ T cell counts between MSI and MSS cancers from African Americans. (C) CD8^+^ T cell counts between MSI and MSS cancers from Caucasians. The number above each dot blot are means; the horizontal bar represents the mean number among the cancers.

## References

[1] Carethers JM, Murali B, Yang B, Doctolero RT, Tajima A, et al (2014) Influence of Race on Microsatellite Instability and CD8^+^ T Cell Infiltration in Colon Cancer. PLoS ONE 9(6): e100461 doi:10.1371/journal.pone.0100461 24956473 10.1371/journal.pone.0100461 PMC4067325

