# Supplementary figures and images for: Influence of Race on Microsatellite Instability and CD8+ T Cell Infiltration in Colon Cancer
Source: PLoS One. 2014 Jun 23;9(6):e100461. doi: 10.1371/journal.pone.0100461 (PMC4067325; doi:10.1371/journal.pone.0100461)

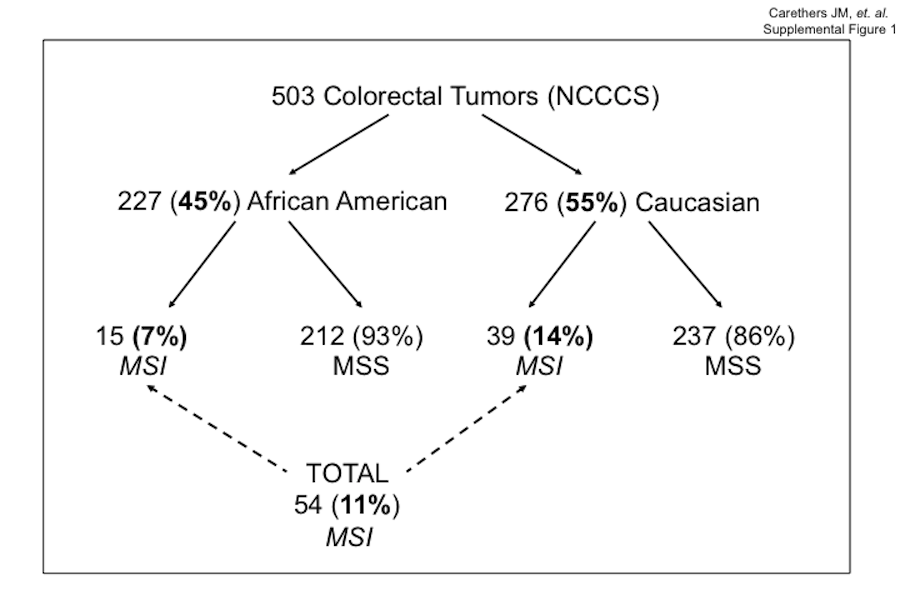

Supplement: Figure S1 — Distribution of MSI and MSS tumors among African American and Caucasian patients in the NCCCS. (TIFF) [file pone.0100461.s001.tif]
